# Targeted Immunology for Prevention and Cure of VL

**DOI:** 10.3389/fimmu.2014.00660

**Published:** 2014-12-19

**Authors:** Nahid Ali, Hira L. Nakhasi, Jesus G. Valenzuela, Alexandre Barbosa Reis

**Affiliations:** ^1^Infectious Diseases and Immunology, Indian Institute of Chemical Biology, Kolkata, India; ^2^US Food and Drug Administration, Silver Spring, MD, USA; ^3^National Institute of Allergy and Infectious Diseases, Rockville, MD, USA; ^4^Universidade Federal de Ouro Preto, Ouro Preto, Brazil

**Keywords:** visceral leishmaniasis, immunotherapy, vaccine, immuno-modulator, Th1 response

Leishmaniasis is a neglected tropical disease caused by a group of protozoan parasites of the genus *Leishmania*. Clinical presentation of leishmaniasis can range from cutaneous, mucocutaneous, or visceral forms depending on the parasite species. Visceral leishmaniasis (VL) caused by *L. donovani* and *L. infantum* is the severest and one of the deadliest parasitic diseases of the tropics second only to malaria (1). Nearly, 20,000–40,000 annual deaths are estimated due to this disease (2). Except for the Indian subcontinent and West Africa, VL is frequent in dogs, which serve as the major reservoir for zoonosis (3).

Transmitted by the bite of an infected sandfly, *Leishmania* endure in the phagolysomal compartment of macrophages by evasion and attenuation of the microbicidal functions of the host (4, 5). *Leishmania* has evolved as a successful parasite chiefly by its ability to modulate the immunological and cyto-chemical responses of the host following infection. The key strategy for successful pathogenesis is to subvert the nitric oxide burst in the host macrophage. This opportunistic parasite thus establishes a safe niche in the inactivated phagocyte and uncontrolled parasitization in liver, spleen, and bone marrow leads to symptomatic VL characterized by fever, weight loss, hepatosplenomegaly, and anemia (3).

Since the pathogenesis of the disease is based on subversion and modulation of both innate and adaptive arms of immunity, the disease is opportunistic to immuno-suppression (6). Hence, commencement of an appropriate immune response is a challenge for the control of VL infection. Indeed, therapeutic drugs like SSG and miltefosine are immuno-modulators that trigger Th1 responses essential for activation of oxidative burst in the macrophages (7, 8). However, major limitations of narrow therapeutic index and increasing incidence of resistance with currently used drugs for VL are encumbrances in effective disease management. Recent approaches like combination therapy, targeted delivery, and use of immune-adjunct are efforts to bring down the effective doses of these toxicity-associated drugs. Most promising are the prospects of various immune-targeted therapeutic approaches for treatment of VL (9). Various leishmanial antigens, cytokines, and antibodies that initiate protective Th1-biased cell-mediated immune responses used singly or as an adjunct to conventional chemotherapy are potent immuno-chemotherapeutic agents for the cure of VL (10, 11). Additionally, medicinal plants and their products have opened new dimensions in search of safer and cheaper anti-leishmanial immuno-modulators. These phytochemicals are not only promising as immuno-chemotherapeutic agents against VL but also have potential as immuno-adjuvants and adjuncts to chemotherapy for a number of other immuno-regulatory diseases (12).

Since both cure and resilience to *Leishmania* infection depend on the immunological status of the host, the antigens that can trigger healing responses can also induce prophylactic immunity. Therefore, identification of immunogens that can induce Th1 responses is the critical aspect of vaccine search against VL (13).

Although a number of defined antigens have been reported to impart protective immunity against experimental VL, recent trend of reverse vaccinology is a promising aspect for identification of key immunogens for a successful vaccine (13, 14). This requires rational inputs and algorithm for identification of a promising antigen from the whole proteome data analysis *in silico* (15). One of the key inputs is to identify the epitopes for activation of both CD4^+^Th1 and CD8^+^ T cells. Indeed, several studies have attempted to generate epitope-based vaccines from potent antigens that selectively targets MHC I and MHC II. These multi-epitope-based synthetic vaccines were found to stimulate Th1 and CD8^+^ T cell responses and can be potentially used for prophylaxis against VL (16, 17). Since antigen presenting cells determine the activation of specific lymphocyte subsets, targeting dendritic cells that are known for activation of Th1 and CD8^+^ T cells can serve as an important vaccination strategy against VL. Indeed, various reports of antigen-pulsed DCs as vaccine against experimental VL have been promising (18).

The vector (sandfly) salivary proteins play a pivotal role in parasite pathogenesis. Indeed, the infective dose of *Leishmania* parasites during natural transmission is much lower as compared to saliva free infectious inoculums (19). This has been primarily attributed to the initial immune responses to salivary component triggered following sandfly bite, which enhances the infectivity of *Leishmania* in the host. Rationally, priming the host against a number of sandfly salivary proteins have been shown to induce altered host immunity to the parasite imparting protection against *Leishmania* infection (20, 21). Therefore, salivary proteins alone or in combination with parasite antigens can be promising vaccine components against VL (22).

However, most defined protein based vaccines are limited by their inability to generate profound long lasting immunity. This is in part due to lack of antigen persistence and multiplicity of antigens required to generate long lasting memory without a suitable adjuvant (23). In fact, lifelong immunity gained through natural infection is the gold standard of protection for VL. Therefore, apart from triggering appropriate immune responses, the immune correlates of long lasting protective immunity have to be determined. This can be achieved in part by the partial mimic of natural infection, which ensures antigen/parasite persistence and multi-antigenicity required for robust long lasting immunity. Although DNA vaccines can ensure antigen persistence, it is limited by multiplicity of antigens required and the potential adverse effects associated.

For this very reason, several genetically modified live parasites have been found to be the most efficient vaccination strategy (23, 24). However, despite reported success as a vaccination strategy against experimental VL, none of the genetically modified organisms have been approved for clinical trials. The primary concern is the safety issue associated with live parasites. Possibility of revert pathogenesis makes the use of live parasites speculative for human administration. However, understanding the biomarkers of safety of the live vaccines in human cell can be highly valuable in the development of a successful vaccine against VL (25).

## Conflict of Interest Statement

The authors declare that the research was conducted in the absence of any commercial or financial relationships that could be construed as a potential conflict of interest.
